# Does *Islamicity* matter for the stability of Islamic banks in dual banking systems?

**DOI:** 10.1016/j.heliyon.2022.e09245

**Published:** 2022-04-06

**Authors:** Abderazak Bakhouche, Teheni El Ghak, Mohammad Alshiab

**Affiliations:** a Business Department, Higher College of Technology, P.O. Box-15825, Dubai campus, United Arab Emirates; b LIEI, Faculty of Economic Sciences and Management of Tunis, University Tunis El Manar, Tunis, Post Box 248 - 2092, Tunis El Manar II, Tunisia; c Business Department, Higher College of Technology, P.O. Box-25035, Abu Dhabi campus, United Arab Emirates

**Keywords:** Islamic banks, Stability, Z-score, Islamicity index

## Abstract

Investigating the relation between the institutional environment and bank stability has become the focus of recent empirical works. Since its emergence, the Islamic segment of dual banking systems has expanded faster than the conventional segment, albeit growth remains somehow impeded due to many factors. In most countries, the business environment is centred on the principle of "*maximisation of owners' wealth*", which may have stripped Islamic banks of their intermediary function to pursue activities in greater congruence with the *alfalah*-*Maqasid Sharia approach* framework. This study examines whether Islamic banks are more stable in countries where the environment is overwhelmed by Islamicity than in countries with less Islamicity. A sample of Islamic and conventional banks from 14 Muslim majority countries is employed for the 2016–19 period. The results suggest that Islamicity has a neutral effect on bank stability and that Islamic banks do not find higher Islamicity of the environment a supporting factor for their resilience. Our findings reject the '*Islamicity-stability*" hypothesis for Islamic banks, suggesting that the Islamicity of the environment is irrelevant in dual banking systems. From a different angle, Islamic banks may seem to be a "disguised" version of conventional banks.

## 1 Introduction

Dual banking systems are characterised by the cohabitation of two separate banking models, i.e. conventional and Islamic. Conventional banking is centuries old and is primarily based on risk-free interest contracts. By contrast, Islamic banking, which is showcased as an alternative to the conventional model (Wasiaturrahma et al., 2020), has only recently emerged due to the Muslims’ demand for an Islamic-*Sharia* compliant mode of banking (Bitar et al., 2020). *Sharia* governs the permissibility of the different aspects of Islamic financial institutions' activities based on two fundamental principles (Sundararajan and Errico, 2002; Cihák and Hesse, 2010; Beck et al., 2013; Abbadi and Silva, 2020). First, banks are prohibited from engaging in usury or *riba*-based transactions which involve charging or earning interest. Second, Sharia promotes risk-sharing models whereby parties in a transaction share gain or loss outcomes. El-Hawary et al. (2004) consider two more characteristics defining Islamic banking and finance: i) no funding of sinful activities and ii) materiality.

The growing importance of faith-based finance in dual banking systems has motivated scholarly investigation of the factors shaping how they behave, how they compete against conventional banks and how they respond to various risks and shocks, such as the 2007-09 global financial crisis (GFC) (Bitar et al., 2020). While a stream of research (e.g. Cihak and Hesse, 2010; Abdulle and Kassim, 2012; Abedifar et al., 2013; Alqahtani et al., 2016) analyses the impact of several bank-specific influences, another stream considers the role played by several environmental factors such as institutional settings (Mollah and Zaman, 2015) and environmental risks (Al-Shboul et al., 2020). However, the results from recent research lead to the consideration that the explaining bank outcomes can be broadened to encapsulate cultural and social forces such as religiosity or religiousness (e.g. Kanagaretnam et al., 2015; Bilgin et al., 2021). For instance, Azmat et al. (2020) and Bilgin et al. (2021) debate whether person religiosity directly or indirectly affects bank risk-taking and stability. Despite the significant results obtained by these studies, the literature has isolated the impact induced by the *Islamicity* of the broader environment on banks’ stability, especially Islamic banks. This issue is highly relevant for Islamic banks because they are expected to operate in a hospitable environment that serves the principal goal of compliance with Sharia dealings. We argue that Islamic banks which operate in environments characterised by higher Islamicity are likely to show stronger resilience and stability.

Research on the relationship between religiosity and bank outcomes in countries with dual banking systems is fairly a recent phenomenon (Kanagaretnam et al., 2015). One segment of this research replicates the works on the impact of personal religiosity on corporate risk-taking in countries not affiliated with the Islamic faith (Hilary and Hui, 2009; Kanagaretnam et al., 2015). It looks at religiosity as a concept reflecting individuals’ varying *propensities* to comply with a set of *faith*-derived beliefs, ethics, principles and practices such as *Sharia* law (Saba et al., 2021). Personal religious beliefs drive personal traits such as ethics, honesty, and organisation behaviours, including agency costs, dividend policy, and corporate risk-taking (McGuire et al., 2012; León and Pfeifer, 2017; Cebula and Rossi, 2021). The results from this literature emphasise the moderating role of religious beliefs. Greater religiosity curbs individual and corporate risk-taking behaviour and more religious individuals have greater risk aversion.

Furthermore, another segment of this research tests the hypothesis that an environment or a system overwhelmed by religiosity impacts bank risk-taking and preferences. For instance, Kanagaretnam et al. (2015) find that banks operating in more religious countries exhibit a lower propensity to engage in risk-taking activities. More recently, Bilgin et al. (2021) evaluate whether economic uncertainty affects Islamic banks' stability differently from conventional banks in Muslim-majority countries. The results indicate that economic uncertainty has a different impact on the stability of both types of banks, confirming evidence for the role of religiosity. First, in countries where religion is important, economic uncertainty negatively affects default risk in conventional banks, but Islamic banks’ default risk is not significantly affected. Second, in countries with a higher share of profit and loss sharing contracts in their national banking systems or implement *Sharia* law, either exclusively or alongside other legal systems, the default risk of Islamic banks in uncertain times is mitigated.

Nevertheless, the existing studies on the bank stability-religiosity nexus in countries with dual banking systems consider a narrower and subjective measurement of individual religiosity. For instance, Kanagaretnam et al. (2015) and Bilgin et al. (2021) use the data availed by World Values Surveys (WVS), which capture different dimensions of time-varying social values and beliefs, including religious orientation. Since 1981, the Surveys collect *representative* data on thousands of respondents from different economies in multiple rounds, which started with 21 countries in the 1981 surveys to 99 countries in the 2005–06 surveys. Kanagaretnam et al. (2015) use proxies of religion variables averaged over the two most World Values Surveys covering the 2000–06 period. Three WVS questions are employed to construct the religiosity variable covering affiliation with a religion (knowledge), the importance of religion (feeling) and observance of religious practices. Bilgin et al. (2021) build a country-level religiosity variable captured by the share of the population who consider religion very important.

Unmistakably, the approach followed by these studies may not unveil the full impact of religiosity because it considers the self-revealed faith of the individuals rather than the extent to which the practices, activities and institutions are guided and compliant with Islamic *Sharia*. Indeed, the datasets from the WVS are a valuable resource for measuring and benchmarking individual religiosity. However, they may be exposed to sampling bias and statistical errors. Additionally, the WVS focuses on person-level religiosity by asking a panel of people in a specific country to rate their commitment to religion or how important religion is in their various aspects of life. It ignores the extent to which the environment consisting of the different economic, political and other institutions incorporates Sharia-sourced injunctions.

This paper extends current research (Kanagaretnam et al., 2015; Bilgin et al., 2021) on how the religiosity of the environment, referred to as *Islamicity*, could affect the stability of Islamic and conventional banks in dual banking systems. To the best of our information, there has not been a study examining the role of *Islamicity* in shaping Islamic banks stability. Broadly, *Islamicity* refers to the degree to which the formal and informal environments align their goals, institutions, structures, practices, and relations with the teachings of the *Sharia* (Askari, 2019). In this sense, *Islamicity* defines the various economic, legal, political and social behaviours and shapes the broad decision-making and risk-taking landscapes where banks operate.

This paper postulates that the stability of Islamic banks is narrowly linked to the degree of *Islamicity* of the broader business environment. It seeks evidence for the assertion that for Islamic banks to contribute to society's banking stability through its stated "*Islamic*" manners, this very society needs to be overwhelmed by the ideals on which Islamic banks are based. This view is shared with Abdul-Baki and Uthman (2017) and Kader (2021), who theorise the ideal environment capable of making Islamic financial institutions reach their inherent potentials. They claim that Islamic banks' societal ends are achieved when the systems in an environment where banks operate share a similar goal of *alfalah*. Therefore, the outcome of this study may help the regulatory authority and banks' decision-makers to understand whether Islamic banks are responsive to a higher “*islamicitised*” environment and are genuinely Islamic as they pretend.

Empirically, to provide insight into the *Islamicity*–bank stability nexus, we employ a dataset comprising Islamic and conventional banks across 14 countries for the period 2016–19. In the regression estimation, stability is measured by the conventional Z-score, while the *Islamicity* of the environment is measured by the novel *Islamicity Index* and its four sub-indices developed by Rehman and Askari (2010). Our methodology is innovative because we use a more objective proxy of the *Islamicity* of the environment rather than the subjective proxies based on the self-avowed *religiosity* of the population. The components of the Islamicity index allow us to further identify the dimensions of *Islamicity* to which banks are most responsive.

In general, the results confirm the literature findings on the impact of personal religiosity on bank stability. The contribution of Islamic banks to banking systems' stability in countries characterised by higher *Islamicity* is as significant as the contribution of their conventional counterparts. Therefore, the *Islamicity* of the environment is irrelevant to the stability of Islamic banks. Islamic banking may be still in the transition stage in their evolution cycle whereby they employ the conventional settings to deal with a rational customer base.

The remainder of this study is organised as follow. Section 2 provides a synopsis of the theoretical framework of this paper. Section 3 provides a brief review of the related literature. Section 4 describes the sample and the methods employed in the empirical analysis. Section 5 presents and discusses the results. Finally, Section 6 concludes and preconises.

## 2 Theoretical framework

In an environment overwhelmed by *Islamicity*, the ultimate goal for individuals, groups, and organisations is to attain *alfalah* (Kader, 2021). It is a concept plenteous in meaning, significations and implications. It refers to utter success, happiness, and accomplishment in this life (*hayat dounya*) and the *Hereafter* (*Akhira*). The life-part of the *alfalah* is closely referred to as individual happiness, social harmony, and economic wellbeing. *Alfalah* incites intentions, attributes, behaviours, relationships and policies in society to embrace goodness, honesty, trust, righteousness, tolerance, candidness, justice, risk-sharing and inclusiveness. By contrast, it loathes dishonesty, injustice, deceit, cheating, misleading, and wealth construction through exploitation (riba), commonly referred to as "*batil*" (Alwi et al., 2021).

Furthermore, there is consensus among *Sharia* scholars that the substance of *Alfalah* can be empirically attained by pursuing the behaviours and policies demonstrated in the *Maqasid Sharia* approach, developed in the 12^th^ century by *Al-Ghazzali*^1^ (Chapra, 2008; Hudaefi and Noordin, 2019; Kader, 2021). *Maqasid Sharia* provides a “*roadmap*” for the permissible *halal* and the prohibited *haram* that Islamic banks apply to develop products and services to alleviate poverty and promote wellbeing (Ibn Ashur, 2016; Alwi et al., 2021; Kader, 2021; Rohman et al., 2021). Like Maslow's Hierarchy of Needs pyramid (1943), the *Al-Ghazzalian* paradigm points out that humankind's *wellbeing* is contingent on preserving five fundamental symbiotic essentials: *al-din*, *al-nafs*, *al-aql*, *al-nasl*, and *al-mal* (Ishak and Asni, 2020; Kader, 2021). We focus on the latter four essentials since *al-din* refers to maintaining worship rituals as per the five pillars of Islam.

First, *al-nafs* refers to life and soul, and its preservation is based on the absolute sanctity of the human body and soul. For the body, this purpose is achieved by ensuring access to basic needs such as food, water, sanitation, health, shelter, and living in a cleaner environment. For the soul, the purpose is accomplished by safeguarding human safety, dignity and rights, and propagating Islamic underpinnings of tolerance, freedom, and justice (Kader, 2021).

Second, *al-aql* is related to intellect, thought and knowledge (Powers, 2004). The preservation of intellect includes protecting mental health, enabling access to education, and promoting other *Sharia*-compliant freedoms.

Third, the preservation of *al-nasl* or progeny and offspring has a relationship with the preservation of family and children. Ibn Ashur^2^ (2016) pinpoints the substantial role of women for this purpose by facilitating their access to adequate health services and educational opportunities.

Fourth, the preservation of *al-mal* or wealth and property refers to the *Sharia*-compliant protection of value and stability. Broadly, this can be achieved through stimulating innovation and entrepreneurship and enabling equal opportunities. It implies setting a business environment where individuals can initiate and materialise entrepreneurial ideas, obtain finance based on Sharia rules, and observe utmost good faith in all stages of contracts, among others.

Taking a cue from the above, *Sharia*-compliant financial institutions are entrusted with preserving the nation's financial resources without exposing them to practices loathed by Sharia (Sarker, 1999; Siddiqui, 2001; Haniffa and Hudaib, 2007; Asutay, 2007; Siddiqi et al., 2019; Khomsatun et al., 2021). Subsequently, Islamic banks contribute to the country's broader attainment of *alfalah-maqasid Sharia* by pursuing the goal of financial stability (Rizvi et al., 2020; Khomsatun et al., 2021). Islamic banks' stability may be more secured if individual behaviours and collective policies prevailing in a country are also overwhelmed by *Islamicity*. In other words, Islamic banks may find it easier to achieve and sustain bank stability if the broader landscape shares the fundamental underpinnings of the Sharia teaching as demonstrated in the *alfalah-maqasid Sharia* approach.

## 3 Literature review

The stability of Islamic banking institutions in dual banking systems has been of interest to academics and policymakers for almost 20 years. The extant literature may be bundled into three strands.

The first strand of the literature evaluates whether the practices of Islamic banks adhere to the conventional tenets of *Sharia* on banking and finance. This research observes that Islamic banks' financial statements are characterised by low profitability and asset portfolios composed mainly of short-term and trade-based transactions (El-Hawary et al., 2004). This structure may be interpreted as that Islamic banks are shifting away from profit and loss sharing activities, which are the *raison d’être* of Islamic banking, for more risk-free conventional transactions (Majeed and Zainab, 2017; Miah and Suzuki, 2020).

One justification of this shift is presented by Kuran (1993), who argues that Islamic banks may have diverged from their risk-sharing based banking model because they are forced to imitate the strategies of their conventional peers. Kuran (1993) explains that banks face an environment characterised by inherent risks driven by widespread asymmetric information and populated by users who are dominated by rational choices. Unlike Islamic banks, the traditional conventional banks have significant advantages in understanding this environment and already have developed the appropriate tools to deal with it. Due to their recent emergence and lacking innovative and sufficiently-tested tools, Islamic banks find themselves forced to employ and replicate conventional practices to endure this deleterious environment and grow further. To survive, Islamic banks are ought to link their metrics to the conventional environment and even remotely follow the standards imposed by conventional banks. As a result, Islamic banks may violate Islamic teachings on banking and finance and display indistinguishable features from conventional banks.

The argument of Kuran (1993) is supported by empirical works such as Khan (2010), who shows that the instruments offered by Islamic banks are mere modifications or imitations of conventional instruments and differ only in their Arabic-coined names. Khan (2010) confirms that Islamic banks violate the fundamental ideals of *Sharia* on banking and finance and supports the view that Islamic banks may not be alternative to conventional banks after all. Miah and Suzuki (2020) find that the operations of Islamic banks are concentrated in debt-like contracts leaving little room for risk-sharing and profit and loss sharing contracts. López Mejía et al. (2014) note that the risk-sharing feature is neutralized as Islamic banks pay competitive “market” returns to investment accounts holders regardless of their performance. Chong and Liu (2009) show that deposit-related operations of Islamic banks are not entirely free of interest practices. Cihak and Hesse (2010) observe that Islamic finance sets profit margins based on a reference rate of return linked to international benchmarks. Mohd and Azha (2019), Nechi and Smaoui (2019) and Saeed et al. (2021) find connectedness between the Sharia-compliant interbank rates and the corresponding conventional interbank offer rates for Gulf Cooperation Council (GCC) countries and Malaysia. Saeed et al. (2021) show that the markets for Islamic investment deposits and conventional fixed deposits are not perfectly segregated.

In contrast, Yousef (2004) argues that the close imitation of practices do not justify the view that Islamic banks are functionally indistinguishable from conventional banks. Majeed and Zainab (2017) find that Islamic banks in Pakistan follow *Sharia* principles, even though they do not provide profit and loss sharing contracts nor Islamic-driven debt contracts. Ahmad (1993) claims that the Islamic banks’ imitation of conventional banks is only one phase in the life cycle of these faith-based banks. Islamic banking is an emerging industry that lacks extensive *Sharia*-compliant human capital resources and appropriate regulatory framework and support. This translates into limited offerings that are fully compatible with *Sharia* dealings. To reach the full-fledged *Islamic* stage, Islamic banks may need to learn traditional banking from the centuries-long existing conventional banks by imitating their practices. Saeed et al. (2021) argue that Islamic banks are forced to benchmark their rates to conventional rates because they face profit-driven customers influenced by rational motivations and economic considerations. In this sense, banks face a trade-off between complete adherence to Islamic Sharia and the reality of economic fundamentals dominated by conventional practices.

The second strand of the literature debates the superiority of Islamic banks over conventional banks in performance, competition, efficiency and stability (Abedifar et al., 2015). Most empirical studies on this debate flourished after the 2007 global financial crisis (GFC) by investigating how both types of banks responded to it. This literature is not harmonious as to whether the Islamicity of a bank enhances its stability. The general lack of consensus across studies may be due to the differences in the analysed bank datasets, countries and periods, and the utilisation of different methodologies and proxies of bank stability.

The advocates of Islamic banking (e.g., Khan, 2010) argue that Islamic banks are more efficient than conventional banks. Ibrahim and Rizvi (2018) show that Islamic banks provide more stability to the banking system by ensuring continuous credit supply during times of difficulty. Rizvi et al. (2020) find that Islamic banks reinforce Indonesia's banking system's stability during a crisis. The advantage of Islamic banks in terms of stability comes from the risk sharing feature (López Mejía et al., 2014), which also promotes allocative efficiency and economic growth (Iqbal and Molyneux, 2005).

Conversely, several studies do not find strong evidence supporting the superiority of Islamic banks in performance and stability. For instance, Abdulle and Kassim (2012) show that the 2007 GFC did not influence the profitability and credit risk of Malaysian Islamic and conventional banks differently. Bourkhis and Nabi (2013) show that the 2007 GFC similarly affected the soundness of Islamic and conventional banks. Studies such as Al-Shboul et al. (2020) and Bilgin et al. (2021) find that Islamic banks’ contribution to stability does not significantly diverge from conventional banks. However, Abdulle and Kassim (2012) find that Islamic banks hold more liquid assets than conventional banks. Alqahtani et al. (2016) show that in the early stages of the 2007 GFC, Islamic banks outperformed their conventional counterparts in the areas of capitalisation, profitability and liquidity. However, in the later stages of the crisis, Islamic banks' capitalisation, profitability, and efficiency deteriorated. Other studies (e.g. López Mejía et al., 2014; Kabir and Worthington, 2017) claim that the role of Islamic banks in the performance and stability of the banking system is overemphasised in the literature as the size of this segment remains small. Abbadi and Silva (2020) warn that the empirical findings on the superiority of Islamic metrics over conventional metrics depends on the dataset under consideration, period of the study, and measures used.

The third strand of literature is a recent phenomenon, and it focuses on the impact of religiosity of the environment on bank performance and stability. This literature originates from the empirical works of Hilary and Hui (2009) and McGuire et al. (2012) on the role of religious beliefs on risk aversion in non-financial corporate culture in non-Muslim countries.

The argument of this literature is based on social identity theories (Tajfel 1978; Islam, 2014), which suggest the value of sharing identity, belonging to a particular group, and conforming to the norms in local culture has a substantial influence on people's behaviours. By taking a cue from this proposition, higher religiosity is associated with lower risk because religiousness is a social mechanism, which moderates individuals' behaviours, social interactions and economic decisions (Li et al., 2013). The degree of religiosity among customers makes them less sensitive to competition, superseding their rationality-based profit maximisation motives (Saeed et al., 2021). Adhikari and Agrawal (2016) show that local beliefs and religiosity disincentive US banks' managers to take on more risks. They find that banks in more religious areas can resist crisis as they exhibit lower default risk, grow their assets more slowly, hold safer assets, rely less on non-traditional banking.

For dual banking systems where Islamic and conventional banks coexist, the literature deliberates whether religiosity contributes to the performance and stability of both types of banks and the overall system. The role of religiosity in shaping bank stability is highlighted in the study of Bilgin et al. (2021), who find that religiosity is more pronounced in Muslim-dominated countries or where religion is viewed as important or Islamic law prevails. They find that the religiosity of the environment shields Islamic banks from default thanks to the profit and loss sharing feature of Islamic banking. Additionally, based on Shu et al. (2012), Islamic religiosity may influence bank offering and risk-taking by affecting managerial decision-making and the country's informal and formal institutions. In principle, the products and services offered by Islamic banks are developed to serve the demands of the populations overwhelmed by Islamic religiosity (Azmat et al., 2020). For this case, banks dominated managers induced by their Islamic religiosity may be more advantageous to understand Sharia's underpinnings and, therefore, be well placed to serve such Islamic populations. This can drive the bank to better comply with Sharia dealings, thus bringing about more robust performance and higher stability. However, this view is not shared with Ali and Azmi (2016), who examine whether boards of directors dominated by members from the Islamic faith can positively impact the performance and stability of both Islamic and conventional banks in Malaysia. The results reveal Islamic orientation is irrelevant to bank performance. Board members from other faiths can manage the Islamic banking business as good as a Muslim board member.

Thus, Islamic banks may be compelled to operate and satisfy two seemingly conflicting environmental settings in dual banking systems. The first setting relates to the *alfalah*-based principles and goals that Islamic banks claim to adhere to and share with their religious customers. Grais and Pellegrini (2006) state that if an Islamic bank does not comply with Sharia, this could erode its reputation and affect its financial resilience. To safeguard bank stability and the interest of the stakeholders, Islamic banks must implement a system of governance compatible with Sharia (Khomasatun et al., 2021). Abdul-Baki and Uthman (2017) assert that Islamic banks achieve their *alfalah*-based goals when the systems where they operate are also motivated by *alfalah* goals. The second setting relates to the GDP-based socio-economic environment overwhelmed by the globalisation-driven goal of "*shareholders wealth maximisation*" based on the "*laissez-faire self-interest”* principle. The importance of conventional structures and practices in global banking and finance and other economic considerations may push Islamic banks to violate Islamic principles.

This paper builds upon the above literature by expanding the concept of religiosity beyond identifying beliefs or faith to the *Islamicity* concept developed by Rehman and Askari (2010). It examines whether the *Islamicity* of the wider environment immunes Islamic banks from default as measured by the Z-score. *Islamicity* upholds individual and institutional honesty and promotes higher disclosure and transparency where agents effectively obtain and exploit dishonesty-free information (e.g. Bushman et al., 2004; Bourgain et al., 2012; Boubakri et al., 2013). Within this context, the *information asymmetries channel* seems to be one mechanism through which the *alfalah*-based environment emphasises bank stability. Abedifar et al. (2013) and Baele et al. (2014) find that borrowers are likely to fulfil their obligations under Islamic loan contracts, maybe due to their strong effect of *Islamicity*.

These studies (e.g. Khomasatun et al., 2021; Bektas et al., 2022) assume that the self-proclaimed adherence to the Islamic religion as published by the WVS is bias-free and, therefore, can capture the impact of religion on bank outcomes. In our paper, we employ the Islamicity index to capture the degree to which the environments where Islamic banks operate incorporates *Sharia* commands.

## 4 Sample, variables and methodology

### 4.1 Sample

To examine the impact of the degree of Islamicity prevailing in an environment as a measure of a society's assumed degree of *Islamicity* or "*alfalahicity*" on the stability of conventional and Islamic banks, we draw data from multiple sources.

First, the bank-level data are extracted from the balance sheets and income statements of the Worldscope database for the period 2016 to 2019 and across 14 countries. We consider countries with dual banking systems where Islamic and conventional banks operate: Bahrain, Egypt, Indonesia, Jordan, Kuwait, Malaysia, Nigeria, Oman, Pakistan, Qatar, Saudi Arabia, United Arab Emirates (UAE), Sri Lanka, and Turkey. Except for Sri Lanka, most of the population in all other countries are Muslims. We retain commercial banks which were listed on their respective national financial markets throughout the period under study. We drop banks with information for fewer than three consecutive years, as the bank stability measures computed in this study are based on rolling windows over the past three years. All bank-year observations for which data are unavailable on retained variables are discarded and dropped (e.g., total assets, total deposit, total net loans).

We finally derive an unbalanced panel of 485 bank-year observations for 51 Islamic and 172 conventional banks. There is a large difference between the number of Islamic and conventional banks. While nearly 23% of our observations relate to Islamic banks, this is similar to previous studies such as Izzeldine et al. (2020). Table 2 presents the distribution of our sample's bank observations and total average assets by operational model and country. The Islamic banking percentage share column reflects the importance of Islamic banking in each country throughout the study. Every country has at least one bank of each type over the period covered.

Second, the Islamicity index as a measure of the degree of compliance of an environment with Sharia principles is retrieved from the Islamicity index foundation.

Third, the country-level macroeconomic data for inflation rates and the GDP growth are drawn from World Development Indicators (WDI) available on the World Bank website.

This paper does not include other institutional variables in our main IV models due to serious multicollinearity concerns^3^. Instead, we only include the variables of financial freedom and governance as instruments.

### 4.2 Dependent variable: Z-score as a proxy of bank stability

The Z-score has been widely employed in the banking literature to proxy bank stability and risk-taking (Schaeck and Čihák, 2014; Ahamed and Mallick, 2019; Wu et al., 2020). It measures the number of standard deviations by which a bank's return has to diminish to exhaust all equity, i.e. becoming insolvent. A lower (higher) Z-score indicates a higher (a lower) exposure to the insolvency risk, and therefore, the bank is more stable (Bourgain et al., 2012).

The Z-score is estimated as follows:

$$\text{Z}-{\text{score}}_{\text{it}}=\frac{{\text{ROA}}_{\text{it}}+{\text{ ​EQTA}}_{\text{it}}}{{\text{ROA}}_{\text{it}}}$$

Where indices i and t denote the bank, and year, respectively; ROA is the return on assets; EQTA denotes the equity to assets ratio; σROA is the standard deviation of ROA. The σROA is calculated over a three-year rolling time window to allow sufficient time variations (Schaeck and Čihák, 2014). We apply the (1+ Z-score) log-transformation to treat data skewness (Laeven and Levine, 2009).

### 4.3 Primary independent variable: Islamicity index and its components

The Islamicity Foundation has compiled the Islamicity Index annually since 2016, based on Rehman and Askari (2010). The index substantiates the extent to which a country's various institutions are congruent with the rules deduced from Islam's foundational teachings.

Askari (2019) provides the intellectual background for the index. It stems from the idea that while the Islamic economic system is market-based, it places greater weights on values: “*greater degree of justice in all aspects of economic management, higher moral standard, honesty and trust exhibited in the marketplace, and all economic transactions, poverty eradication, a more even distribution of wealth and income, no hoarding of wealth, no opulence in consumption, no exploitive speculation, risk-sharing as opposed to debt contracts, better social infrastructure and provision of social services, better treatment of workers, higher education expenditures relative, a higher degree of environmental preservation, and vigilantly supervised markets*” (Askari, 2019, p. 4).

The index employs multifaceted indicators to paint a panorama of how closely a country embraces policies based on the Quran and Sunnah. It ranges within the bounds of [0.10], with 10 indicating the highest level of Islamicity. The 2019 iteration includes 151 countries, with values ranging from 1.06 to 9.07.

**Figure 1:**
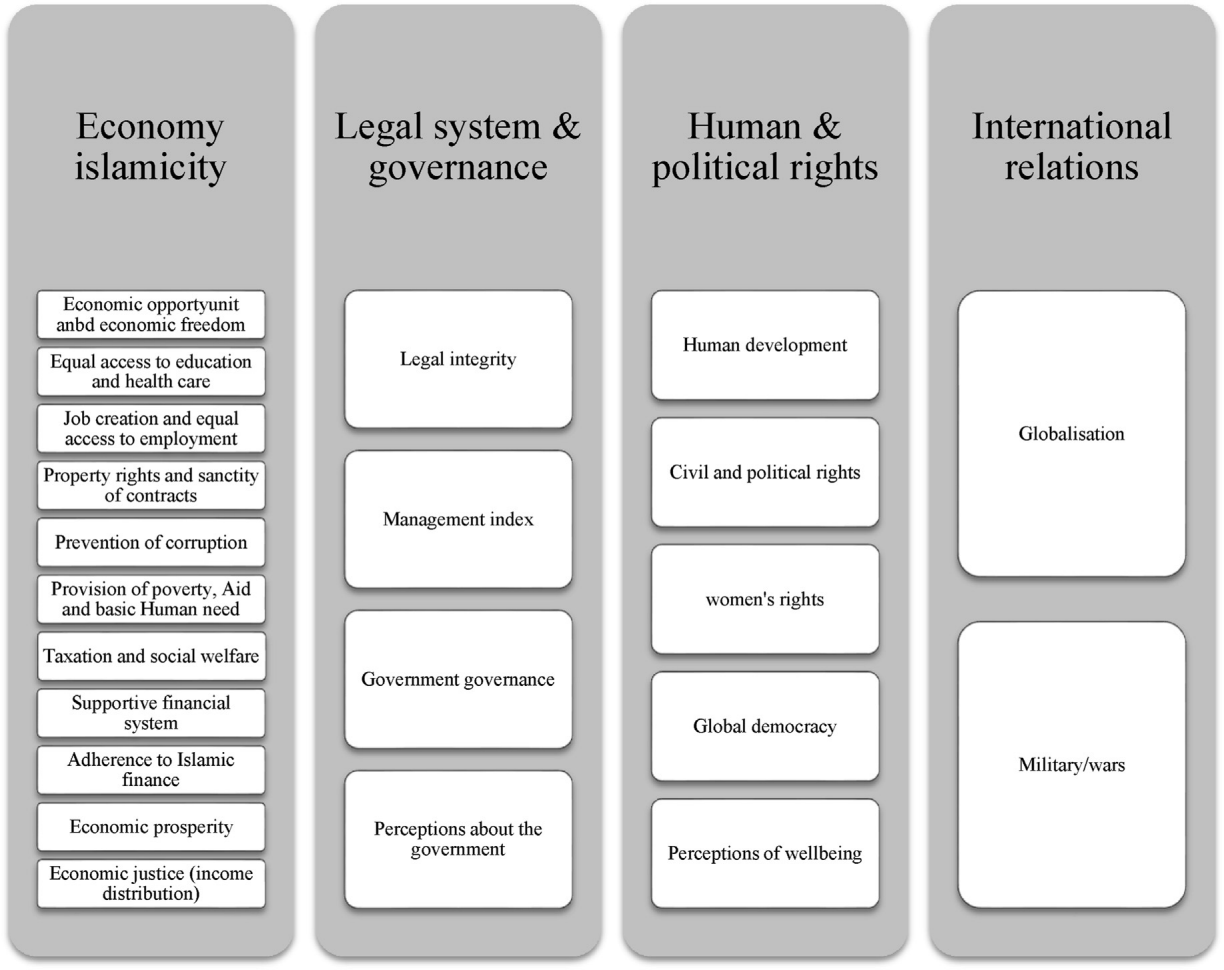
Dimensions and elements of the Islamicity index. Source: Islamicity Indices and their Elements, available at: http://islamicity-index.org/wp/links-downloads/index-elements/.

The index includes four broad dimensions of a Muslim community based on teachings from the Quran—economy Islamicity, legal system and governance, human and political rights, and international relations (Hossein and Mohammadkhan, 2016). A separate sub-index is constructed for each dimension. The sum of all of the four sub-indices forms the Overall Islamicity Index. Figure 1 details the dimensions and elements used to compile the Islamicity Index.

Each column lists the elements used to construct each of the four broad dimensions of Islamicity on which the Islamicity Index is based: economy, legal system & governance, human & political rights, and international relations. The boxes below each dimension identify its underlying components^4^.

First, consider the economy Islamicity dimension, which according to the conceptual framework for the Islamicity Index, focuses on whether the economic and financial systems are compatible with the market-based Islamic system. The elements of this dimension include components of access to basic human needs, individual and societal welfare and opportunity: economic freedom, fair income distribution, preventing poverty, access to education and employment, economic stability, adherence to Islamic finance. The indicators used to measure this dimension includes, among others, gender equality index, ease of doing business, economic freedom index, transparency index, poverty effectiveness, tax burden as a proportion of GDP, social welfare, investment freedom, financial freedom, monetary freedom, financial market risk freedom, absence of interest, macroeconomic indicators, inflation, and economic performance.

The second dimension of the Islamic Index, legal system & governance, considers whether the existing institutions promote sound management of natural resources, environmental quality, the integrity of the legal system, sound governance and management of the country according to Islamic ways. The elements of this dimension include legal integrity, management index, governance and perceptions about the government. Among the indicators used to build this dimension is the protection of human rights index, business costs of crime, the rule of law, country management index, air quality, protection of animal rights, political stability, absence of violence, control of corruption, and trust in government.

The third dimension of the Islamicity Index is human and political rights, which focuses on whether the country can provide basic needs and prepare sound conditions for individuals to develop their welfare and exploit opportunities. Components of this dimension include human development measured by human development index, civilian and political rights, women's rights measured by the proportion of seats held by women in national parliament, global democracy proxied by global democracy index, and perceptions of wellbeing measured by standards of living and safety, freedom of choice, and overall life satisfaction. There are two components in this dimension: globalisation and militarisation and wars.

The final dimension of the index is international relations. It considers whether the country adopts a foreign policy and holds relations with other countries based on Islamic values of peace and respect. Globalisation is measured by the economic globalisation indicator, social globalisation indicator, freedom of foreigners to visit, political globalisation indicator. The militarisation index measures the wars and militarisation.

It can be observed that several elements considered in the Islamicity index resemble the social progress index compiled by the social progress imperative (Boulton, 2021). Rahman (2019) recognises the difficulty in capturing the “*islamicness*” of each dimension as there is a lack of indicators of *islamicness* character. The index relies on readily available indicators but serves conventional purposes that overlap with Islamic principles.

### 4.4 Bank-level controls

We consider six bank-level variables to capture the effects of bank structure and business model on bank stability. Numerous studies find these variables to explain bank stability and risk (Mirzaei and Aguir, 2020; Bhowmik and Sarker, 2021).

#### 4.4.1 Bank size

We consider the natural logarithm of total assets to control for size and economies of scale (. We anticipate larger banks to exhibit relatively better stability (Schaeck and Čihák, 2014). The ratio of total loans over total bank deposits is employed to capture the impact of bank liquidity (Shim, 2019). A higher (lower) value implies higher (lower) bank liquidity risk (Bourgain et al., 2012; Danisman et al., 2021; Mirzaei and Aguir, 2020).

#### 4.4.2 Liquidity

We capture the degree of bank liquidity by incorporating the ratio of total liquid assets (cash and marketable securities) to total assets (Shim, 2019).

Liquidity captures the bank's ability to meet short-term financial obligations without having its investments or fixed assets sold quickly at lower prices. Banks with higher liquidity ratios are expected to be more resilient to financial shocks. Liquidity assets play a mitigating role by enabling banks to meet unexpected withdrawals (Bourgain et al., 2012). Several financial institutions failed because they lacked adequate liquidity levels during the 2007 GFC. Berger and Bouwman (2013) and DeYoung and Torna (2013) show that holding more liquid assets can reduce liquidity risk and help banks lower the likelihood of failure.

Thus, liquidity is expected to be positively related to a bank's financial stability. A higher (lower) value implies higher (lower) bank liquidity risk (Mirzaei and Aguir, 2020).

#### 4.4.3 Assets growth

Assets growth is included to account for differences in risk preference (Schaeck and Čihák, 2014). It is the annual percentage change in total assets. Rapid growth is likely to yield relatively high fragility (Mirzaei and Aguir, 2020). Higher growth may reflect risk-taking behaviour (Bilgin et al., 2021).

#### 4.4.4 Diversification

Diversification is proxied by the income diversification ratio (Laeven and Levine, 2009) computed as:

$$\text{DIV}=\frac{{\text{Net ​interest ​income}}_{\text{it}}-{\text{ ​other ​operating ​income}}_{\text{it}}}{{\text{total ​operating ​income}}_{\text{it}}}$$

Based on (Schaeck and Čihák, 2014), we assume that diversification is positively associated with bank stability. Bilgin et al. (2021) find that diversification is associated with higher risk-taking.

#### 4.4.5 Cost efficiency

Inefficient banks are expected to be more prone to financial fragility (Mirzaei and Aguir, 2020; Bilgin et al., 2021). We estimate efficiency from a bank production specification based on the intermediation approach proposed by Sealey and Lindley (1977), which considers that banks are financial intermediaries that incur different costs when they purchase input to transom into earning assets. The following translog functional form is used to model the underlying cost structure of banks in our sample.

(1) $${\text{EFF}}_{\text{it}}=\ln\left(\frac{{\text{TC}}_{\text{it}}}{{\text{W}}_{3\text{it}}}\right)={\text{α}}_{0}+{\text{α}}_{1}\;\ln{\text{Q}}_{1\;\text{it}}+{\text{α}}_{2}\;\ln{\text{Q}}_{2\;\text{it}}+{\text{β}}_{1}\;\ln\left(\frac{{\text{W}}_{1\text{it}}}{{\text{W}}_{3\text{it}}}\right)+{\text{β}}_{2}\;\ln\left(\frac{{\text{W}}_{2\text{it}}}{{\text{W}}_{3\text{it}}}\right)+\frac{1}{2}{\text{α}}_{11}{\left(\ln{\text{Q}}_{\text{i}1\text{t}}\right)}^{2}+{\text{α}}_{22}\frac{1}{2}\left(\ln{\text{Q}}_{2\text{it}}\right)+\frac{1}{2}{\text{β}}_{11}{\left(\frac{{\text{W}}_{1\text{it}}}{{\text{W}}_{3\text{it}}}\right)}^{2}+\frac{1}{2}{\text{β}}_{22}{\left(\frac{{\text{W}}_{2\text{it}}}{{\text{W}}_{3\text{it}}}\right)}^{2}+{\text{β}}_{12}\;\ln\left(\frac{{\text{W}}_{1\text{it}}}{{\text{W}}_{3\text{it}}}\right)\ln\left(\frac{{\text{W}}_{2\text{it}}}{{\text{W}}_{3\text{it}}}\right)+{\text{β}}_{111}\;\ln{\text{Q}}_{1\;\text{it}}\;\ln\left(\frac{{\text{W}}_{1\text{it}}}{{\text{W}}_{3\text{it}}}\right)+{\text{β}}_{112}\;\ln{\text{Q}}_{2\;\text{it}}\;\ln\left(\frac{{\text{W}}_{1\text{it}}}{{\text{W}}_{3\text{it}}}\right)+{\text{β}}_{121}\;\ln{\text{Q}}_{1\;\text{it}}\;\ln\left(\frac{{\text{W}}_{2\text{it}}}{{\text{W}}_{3\text{it}}}\right)+{\text{β}}_{122}\;\ln{\text{Q}}_{2\;\text{it}}\;\ln\left(\frac{{\text{W}}_{2\text{it}}}{{\text{W}}_{3\text{it}}}\right)+\text{ln}{\text{υ}}_{\text{it}}\text{ ​}+\text{ ​ln}{\text{μ}}_{\text{it}}$$

where TC is the operating cost (the sum of interest expenses, personnel expense and noninterest expense); Q1 is net loans; Q2 is investment securities; W1 is the price of purchased loans (interest expenses to total purchased funds ratio); W2 is the price of labour (personnel expenses to total assets ratio); W3 is the price of physical capital (other noninterest expense to fixed assets ratio); υ_it_ are identical and independently distributed random variables, which are independent of the μ_it_, which are non-negative random variables that are assumed to account for inefficiency (Williams, 2012).

#### 4.4.6 Competition: Lerner of market power

We use the Lerner index of market power to capture competition. The index can be estimated at the bank level for each period (Miah et al., 2020). The Lerner Index is computed as follows:

(2) $${\text{LERNER}}_{\text{it}}=\frac{P_{it}-MC_{it}\;}{P_{it}}$$

Where *i* and *t* denote bank and year, respectively; P is the price of outputs measured by the ratio of total income over total assets; MC is marginal costs. Lerner values range from 0 to 1, with larger values implying greater market power and less competition.

If Lerner is positively related to the Z-score of bank stability, this is consistent with the "competition-fragility" view. This view suggests that higher competition reduces banks' charter value and incentives to behave prudently (Keeley, 1990; Hellmann et al., 2000; Repullo, 2004; Cubillas and González, 2014).

We derive the marginal cost MC from the following translog cost function:

(3) $$\ln\left(\frac{{\text{TC}}_{\text{it}}}{{\text{W}}_{3\text{it}}}\right)={\text{α}}_{\text{0}}+{\text{α}}_{1}\;\ln{\text{TA}}_{\text{it}}+{\text{β}}_{1}\;\ln\left(\frac{{\text{W}}_{1\text{it}}}{{\text{W}}_{3\text{it}}}\right)+{\text{β}}_{2}\;\ln\left(\frac{{\text{W}}_{2\text{it}}}{{\text{W}}_{3\text{it}}}\right)+\frac{1}{2}{\text{α}}_{11}{\left({\text{lnTA}}_{\text{it}}\right)}^{2}+\frac{1}{2}{\text{β}}_{11}{\left(\frac{{\text{W}}_{1\text{it}}}{{\text{W}}_{3\text{it}}}\right)}^{2}+\frac{1}{2}{\text{β}}_{22}{\left(\frac{{\text{W}}_{2\text{it}}}{{\text{W}}_{3\text{it}}}\right)}^{2}+{\text{β}}_{12}\;\ln\left(\frac{{\text{W}}_{1\text{it}}}{{\text{W}}_{3\text{it}}}\right)\ln\left(\frac{{\text{W}}_{2\text{it}}}{{\text{W}}_{3\text{it}}}\right)+{\text{β}}_{111}{\text{lnTA}}_{\text{it}}\;\ln\left(\frac{{\text{W}}_{1\text{it}}}{{\text{W}}_{3\text{it}}}\right){\text{β}}_{112}{\text{lnTA}}_{\text{it}}\;\ln\left(\frac{{\text{W}}_{2\text{it}}}{{\text{W}}_{3\text{it}}}\right)+\ln{\text{υ}}_{\text{it}}+\ln{\text{μ}}_{\text{it}}$$

where TC is the total interest and noninterest expenses; TA is the output (total assets); W is a vector of input prices (price of purchased funds, price of labour, price of physical capital) where: W_1_ is the price of purchased funds calculated as the ratio of total interest expenses to total purchased funds, W_2_ is the price of labour computed as the ratio of total labour expenses to total assets, W_3_ is the price of physical capital: calculated as the ratio of other noninterest expenses to the fixed asset; υ represents standard statistical noise, and μ captures inefficiency. The total costs and prices of funds and labour are scaled by physical capital's price (W_3_) to correct for heteroscedasticity and scale biases (Turk-Ariss, 2010).

Marginal cost is then estimated as (Risfandy et al., 2010):

(4) $${\text{MC}}_{\text{it}}=\frac{\partial {\text{lnTC}}_{\text{it}}}{\partial {\text{lnA}}_{\text{it}}}=\frac{{\text{TC}}_{\text{it}}}{{\text{A}}_{\text{it}}}\left[{\text{α}}_{1}+{\text{α}}_{2}\;\ln{\text{TA}}_{\text{it}}+{\text{β}}_{111}\text{ln}\left(\frac{{\text{W}}_{1\text{it}}}{{\text{W}}_{3\text{it}}}\right)+{\text{β}}_{112}\text{ln}\left(\frac{{\text{W}}_{2\text{it}}}{{\text{W}}_{3\text{it}}}\right)\right]$$

### 4.5 Economic conditions

We control for the impact of the economic conditions and business cycles on bank stability. Albertazzi and Gambacorta (2009), Jokipi and Monnin (2013) and Chen et al. (2017) find that a bank's risk is influenced by business cycles. Poor economic conditions may worsen loan quality, resulting in credit losses and reduced profits. Bank financial stability (e.g. problem loans) usually develops according to the business cycle (Mirzaei and Aguir, 2020).

Similar to Bourgain et al. (2012), we consider GDP growth and inflation. First, the GDP growth rate describes the impact of business cycles on bank performance. We expect that GDP per capita growth is positively associated with higher soundness of banks and less bank fragility (Al-Shboul et al., 2020).

Second, inflation is measured by the percentage change in the consumer price index. Caglayan and Xu (2016) found that inflation volatility severely affects bank loans allocation and, therefore, affects bank riskiness. Adrian and Shin (2009) stated that a higher inflation rate raises the collateral price, thus changing banks' risk perception and making them more risk-tolerant. For Bilgin et al. (2021), higher inflation is associated with riskiness because it reflects deteriorating economic conditions.

Table 1 presents variable definitions and data sources.

**Table 1:** Variables explantations.

Variables	Explanation	Primary references in the literature	Data Sources
**Dependent variables**			
Z-score	Log of Z-score to measure bank solvency risk and stability.	(Kabir and Worthington, 2017) and (Mirzaei and Aguir, 2020)	Worldscope, authors calculation
Islamicity			
OIS	Overall Islamicity index		Rehman and Askari (2010)
EI	Economic Islamicity index.		Rehman and Askari (2010)
LSG	Legal system and governance.		
HPR	Human and political rights.		
IR	International relations.		
**Bank control variables**			
LERNER	Lerner index of market power	Miah et al. (2020)	Worldscope, authors calculation
EFF	A measure of cost efficiency.	Miah et al. (2020)	
DIV	An income diversification index.	Schaeck and Čihák (2014); Mirzaei and Aguir (2020)	
LIQUIDITY	Total liquid assets to total assets		
SIZE	Bank's total assets (log).	Mirzaei and Aguir (2020)	
GROWTH	Annual growth of total assets.		
**Country-level variables**			
GDPGOWTH	Real GDP growth (log).	Mirzaei and Aguir (2020)	World Bank website from world development indicators (WDI) reports
INFLATION	Changes in GDP price deflator		

### 4.6 Econometric model

To investigate the impact of the degree of Islamicity prevalent in an environment on the stability of conventional and Islamic banks, we rely on the literature on the links between environmental variables and bank stability in deriving the econometric equation, such as Bourkhis and Nabi (2013), Al-Shbool et al. (2020) and Boulton (2021). We propose the following empirical specification:

(5) $$\text{Z}-{\text{score}}_{\text{it}}={\text{α}}_{0}+{\text{α}}_{1}\text{Z}-{\text{score}}_{\text{it}-1}+{\text{α}}_{2}{\text{IS}}_{\text{jt}}+\overset{6}{\underset{\text{k}=1}{\sum }}{\text{γ}}_{\text{k}}{\text{BC}}_{\text{it}}+\overset{2}{\underset{\text{m}=1}{\sum }}{\text{δ}}_{\text{m}}{\text{Z}}_{\text{jt}}+{\text{ε}}_{\text{ijt}}$$

Where indices i, j, t denote the bank, country, and year, respectively; Z-score is the dependent variable representing bank stability and risk-taking behaviour; IS represents Islamicity Indices (or its dimensions); ${\text{BC}}_{\text{ijt}}$ is a set of bank control variables; Z is a vector of country-level macroeconomic variables; $\varepsilon_{ijt}$ is the error term assumed to be normally distributed; that is, ${\text{ε}}_{\text{ijt}}∼\text{iid ​N}\left(0,{\text{σ}}^{2}\right)$.

We extend the baseline regression model specified in Eq. (5) to incorporate the dummy variable *ISLAMIC* to identify the relationship between Islamic banks and stability. Therefore, Eq. (5) is extended into the following equation:

(6) $$\text{Z}-{\text{score}}_{\text{it}}={\text{α}}_{0}+{\text{α}}_{1}\text{Z}-{\text{score}}_{\text{it}-1}+{\text{α}}_{2}{\text{IS}}_{\text{jt}}+{\text{α}}_{3}{\text{ISLAMIC}}_{\text{i}}+\overset{6}{\underset{\text{k}=1}{\sum }}{\text{γ}}_{\text{k}}{\text{BC}}_{\text{ijt}}+\overset{2}{\underset{\text{m}=1}{\sum }}{\text{δ}}_{\text{m}}{\text{Z}}_{\text{jt}}+{\text{ε}}_{\text{ijt}}$$

Where *ISLAMIC* is a dummy variable equal to 1 for banks that are Islamic, 0 otherwise; is a set of bank control variables.

For the next stage of our estimation, we extend the regression model specified in Eq. (6) to examine the paper's central hypothesis, which states that Islamic banks are likely to be more stable in an environment dominated by *Islamicity*. Therefore, the model is:

(7) $$\text{Z}-{\text{score}}_{\text{ijt}}={\text{α}}_{0}+{\text{α}}_{1}\text{Z}-{\text{score}}_{\text{ijt}-1}+{\text{α}}_{2}{\text{IS}}_{\text{jt}}+{\text{α}}_{3}{\text{ISLAMIC}}_{\text{i}}+{\text{α}}_{4}{\text{IS}}_{\text{jt}}x{\text{ ​ISLAMIC}}_{\text{i}}+\overset{6}{\underset{\text{k}=1}{\sum }}{\text{γ}}_{\text{k}}{\text{BC}}_{\text{ijt}}+\overset{2}{\underset{\text{m}=1}{\sum }}{\text{δ}}_{\text{m}}{\text{Z}}_{\text{jt}}+{\text{ε}}_{\text{ijt}}$$

Where the interaction term " $IS_{jt}xISLAMIC_{i}$" is an interaction term between the Islamic Bank dummy variable and the Islamicity index (and its dimensions). The coefficient of the interaction term (α_4_) measures the effect of the Islamicity index on the difference in stability between Islamic and conventional banks. A positive (negative) coefficient means that Islamicity has a greater (lesser) magnifying effect on the stability of Islamic banks than that of conventional banks. If the coefficient is found insignificant, then conventional and Islamic banks do not differ in their banking practices, and both benefit from Islamicity, and that environment is homogenous for both. The magnitude of the effects of the Islamicity index on conventional banks can be detected by the coefficient of α_3_, while for Islamic banks, the effect is measured by the coefficient (α_3_ + α_4_).

Our analysis's modelling procedures are congruent with (Cubillas et al., 2020). The panel two-stage least-squares - instrumental variables (IV-2SLSV) method is used to deal with the possible endogeneity in reverse causality. For the potential endogeneity of the variables, we obtain the GMM C statistic test for exogeneity. The null hypothesis is that the variables are exogenous. The Hansen's J test is used to assess the validity of instruments.

## 5 Empirical results

### 5.1 Descriptive statistics

Table 2 presents the descriptive statistics of variables of all banks in the sample. We report the values of the mean, median, standard deviation, and the second and third quartiles. The quantities in Tables 3, 4, and 5 are not expressed in logarithmic terms. Total asset as a proxy of size is held in millions of USD.

**Table 2:** Descriptive statistics for all banks.

	Mean	median	St. dev	Perc. 25^th^	Perc. 75^th^
Number of obs: 257					
Z-score	88.27	58.29	117.39	32.70	105.34
SIZE	22,810.88	8,980.76	35,520.37	3,672.52	25,136.12
GROWTH	0.06	0.05	0.16	-0.01	0.13
LIQUIDITY	0.09	0.08	0.05	0.06	0.11
DIVERSIFICATION	0.17	0.16	0.09	0.11	0.23
EFFICIENCY	0.74	0.77	0.14	0.67	0.84
LERNER	0.33	0.33	0.11	0.25	0.40
INFLATION	0.00	0.01	0.08	-0.03	0.04
GDPGROWTH	0.01	0.01	0.03	-0.01	0.03
OIS	4.51	4.74	1.29	4.06	5.10
EI	5.33	5.57	1.50	4.35	6.58
LSG	4.74	4.99	1.80	4.31	5.69
HPR	3.95	4.18	1.36	3.44	4.81
IR	3.92	3.82	1.53	2.86	4.87

**Table 3:** Mean values for the variables per year and for Islamic and conventional banks.

	Panel 1: Years				Panel 2: Islamic vs conventional	
	2016	2017	2018	2019	Islamic banks	Non-islamic banks
Number of obs	168	172	169	148	191	466
Z-score	88.44	88.05	87.80	88.89	86.51	88.99
SIZE	21,207.13	22,556.05	23,362.17	24,298.02	19,983.38	23,969.80
GROWTH	0.03	0.10	0.02	0.10	0.07	0.06
LIQUIDITY	0.09	0.09	0.09	0.09	0.11	0.08
DIVERSIFICATION	0.18	0.17	0.16	0.15	0.18	0.16
EFFICIENCY	0.73	0.74	0.74	0.74	0.72	0.73
LERNER	0.35	0.33	0.32	0.31	0.33	0.28
INFLATION	-0.04	0.00	0.03	-0.01	0.00	-0.00
GDPGROWTH	0.01	0.01	0.01	0.00	-0.00	0.01
OIS	4.69	4.28	4.53	4.54	4.36	4.49
EI	5.48	5.03	5.35	5.49	5.28	5.26
LSG	4.86	4.47	4.75	4.92	4.58	4.80
HPR	3.85	4.47	3.76	3.69	3.79	4.02
IR	4.34	3.87	3.78	3.64	3.65	3.95

**Table 4:** Mean values for the variables per country.

Country	No. obs	Z-score	SIZE	GROWTH	LIQUIDITY	DIVERSIFICATION	EFFICIENCY	LERNER	INFLATION	GDPGROWTH	OIS	EI	LSG	HPR	IR
Bahrain	36	111.20	14,003.66	0.04	0.08	0.18	0.76	0.29	0.03	-0.02	4.99	6.72	5.02	4.40	2.52
Egypt	46	32.04	5,343.95	0.03	0.07	0.10	0.81	0.40	-0.07	0.03	2.20	2.56	2.28	1.84	2.04
Indonesia	108	84.46	15,535.03	0.11	0.10	0.11	0.60	0.32	0.02	0.04	4.88	5.35	4.87	4.34	5.84
Jordan	46	64.85	7,808.91	0.05	0.13	0.15	0.75	0.38	0.02	-0.00	4.64	5.41	5.51	4.15	3.01
Kuwait	40	137.74	26,013.81	0.09	0.06	0.16	0.81	0.34	0.04	-0.02	4.97	5.97	5.13	4.55	3.66
Malaysia	28	309.20	70,227.24	0.03	0.06	0.27	0.77	0.19	0.00	0.03	6.18	7.21	6.61	5.25	5.93
Nigeria	45	59.43	9,169.68	-0.02	0.19	0.26	0.59	0.31	-0.05	-0.02	2.81	3.31	1.60	2.22	5.69
Oman	32	79.36	9,176.08	0.12	0.08	0.18	0.80	0.31	0.01	-0.02	5.13	6.15	5.95	4.49	3.21
Pakistan	67	36.12	7,814.63	0.05	0.08	0.13	0.76	0.28	-0.03	0.02	2.56	3.69	2.03	1.85	2.72
Qatar	32	134.45	53,491.65	0.04	0.05	0.16	0.89	0.34	0.01	-0.02	5.91	6.86	7.06	5.18	4.27
Saudi Arabia	44	115.01	53,691.58	0.06	0.06	0.25	0.76	0.39	0.04	-0.01	4.57	5.58	5.28	4.03	2.55
Sri Lanka	36	64.27	3,247.56	0.08	0.07	0.11	0.79	0.36	-0.02	0.02	4.67	4.69	4.81	4.96	3.59
Turkey	27	39.36	42,152.01	-0.01	0.11	0.16	0.77	0.21	-0.07	0.02	4.36	4.95	4.56	4.12	2.70
UAE	70	83.91	37,626.58	0.08	0.10	0.23	0.76	0.40	0.02	0.01	6.15	7.36	7.25	5.20	4.49

**Table 5:** Regression results for Islamicity and bank stability for the sample.

	Column 1: OIS	Column 2: EI	Column 3: LSG	Column 4: HPR	Column 5: IR
Constant	-0.6769 (0.203)∗∗∗	-0.7243 (0.1914)∗∗∗	-0.6849 (0.1989)∗∗∗	-0.6806 (0.2042)∗∗∗	-0.7231 (0.230)∗∗∗
Z-score_t-1_	0.9628 (0.0152)∗∗∗	0.9605 (0.0151)∗∗∗	0.9615 (0.0152)∗∗∗	0.9628 (0.0152)∗∗∗	0.9726 (0.0217)∗∗∗
IS	-0.0246 (0.0237)	-0.0175 (0.0216)	-0.0191 (0.0167)	-0.0234 (0.0251)	-0.0459 (0.0639)
EFFICIENCY	-0.0697 (0.0275)∗∗	-0.0652 (0.0281)∗∗	-0.0592 (0.029)∗∗	-0.0664 (0.0279)∗∗	-0.1645 (0.1298)∗∗
LERNER	0.4381 (0.0893)∗∗∗	0.4203 (0.0857)∗∗∗	0.4772 (0.1019)∗∗∗	0.4469 (0.0941)∗∗∗	0.4026 (0.0971)∗∗∗
DIV	0.5626 (0.2196)∗∗	0.5928 (0.2295)∗∗	0.5236 (0.2164)∗∗	0.5615 (0.2227)∗∗	0.7246 (0.3761)∗∗
SIZE	0.0189 (0.0075)∗∗	0.0188 (0.0081)∗∗	0.0209 (0.0084)∗∗	0.017 (0.007)∗∗	0.0124 (0.0076)∗∗
GROWTH	0.0118 (0.0375)	0.0071 (0.0383)	0.016 (0.0377)	0.0201 (0.0386)	0.0313 (0.0474)
LIQUIDITY	-0.3026 (0.1406)∗∗	-0.2974 (0.1485)∗∗	-0.32 (0.1464)∗∗	-0.3018 (0.146)∗∗	-0.1284 (0.1891)∗∗
INFLATION	0.0025 (0.087)	-0.0122 (0.0921)	-0.0071 (0.072)	-0.002 (0.0914)	0.0284 (0.1593)
GDPGOWTH	0.0058 (0.2007)	-0.0515 (0.209)	-0.0555 (0.205)	-0.0419 (0.2135)	0.7358 (1.0679)
Number of obs.	485	485	485	485	485
Wald test (p-value)	0.000	0.000	0.000	0.000	0.000
R-squared	0.9442	0.9453	0.9441	0.9424	0.9325
Root MSE	0.12687	0.12564	0.12695	0.12887	0.13949
Hansen's J test (p-value)	0.3841	0.2664	0.4635	0.3407	0.3439
GMM C statistic (p-value)	0.4399	0.6945	0.4134	0.3201	0.4093

Standard errors in ​brackets. ∗Statistical significance at 10 per cent.∗∗Statistical significance at 5 per cent.∗∗∗Statistical significance at 1 per cent.

Five variables present Islamicity: the overall Islamicity index (OIS) and the four dimensions of the overall index: Economy Islamicity Index (EI), Legal and Governance Index (LSG), Human and Political Rights Index (HPR), and International Relations Index (IR).

For the main variable, the mean value of the Z-score is 88.2 (4.48 in log terms) with a standard deviation of 117.39 (3.77 in log terms).

Our results in Panel 1 in Table 3 indicate that during the 2016–19 period, the average bank in the sample expanded in size. However, it experiences deterioration in the values of diversification and Lerner. For efficiency and liquidity, values remained stable. Further, our results in Panel 2 in Table 3 show that Islamic banks do not differ from conventional banks in terms of stability. The Z-score for conventional banks is 88.99 (log of 4.49), whereas, for Islamic banks, Z-score is 86.51 (log of 4.46). This result contrasts with Miah et al. (2020), who show Islamic banks to be more stable than conventional banks. As expected, Islamic banks are smaller but superior in assets growth, liquidity and income diversification compared to conventional banks. Additionally, Islamic banks (0.33) have higher Lerner values than conventional banks (0.28). For efficiency, both types of banks have a similar average value of 0.74.

Tables 2 and 4 show that the average Islamicity Index among the sample countries is 4.51, ranging from 2.20 for Egypt to 6.15 for the UAE. While Panel 1 in Table 3 shows that Islamicity values did not vastly change between 2016 and 2019, Panel 2 indicates that conventional banks operate in a system subjected to higher Islamicity than Islamic banks. Among all the sub-indices, the Economic Islamicity index has the highest value.

For macroeconomic indicators, the average GDP growth is 1% among the sample countries and ranges between -2% for Qatar, Nigeria and Bahrain to 4% for Indonesia. For inflation, while some countries such as Egypt, Nigeria and Turkey experience negative rates, other countries such as Kuwait and Saudi Arabia witnessed higher rates.

### 5.2 Regression results: effect of Islamicity on bank stability

Tables 5, 6, and 7 present the 2SLS regression results from the models specified in Eqs. (5), (6), and (7). These regressions take the Z-score as the bank-level dependent variable to test the Islamicity-stability link in markets that adopt a dual banking arrangement.

**Table 6:** Regression results for Islamicity, bank stability for our sample, and role of Islamic banking.

	Column 1: OIS	Column 2: EI	Column 3: LSG	Column 4: HPR	Column 5: IR
Constant	-0.6699 (0.2025)∗∗∗	-0.7167 (0.1914)∗∗∗	-0.6779 (0.1986)∗∗∗	-0.6728 (0.2035)∗∗∗	-0.7276 (0.2216)∗∗∗
Z-score_t-1_	0.9622 (0.0152)∗∗∗	0.9595 (0.015)∗∗∗	0.9607 (0.0151)∗∗∗	0.9621 (0.0152)∗∗∗	0.9719 (0.0207)∗∗∗
IS	-0.0252 (0.0242)	-0.0173 (0.0221)	-0.0193 (0.0169)	-0.0241 (0.0257)	-0.0416 (0.0512)
ISLAMIC	0.0083 (0.0137)	0.0111 (0.013)	0.0099 (0.0129)	0.0082 (0.0145)	-0.0029 (0.0257)
EFFICIENCY	-0.0693 (0.0275)∗∗	-0.065 (0.0281)∗∗	-0.0586 (0.029)∗∗	-0.066 (0.0279)∗∗	-0.156 (0.1065)
LERNER	0.4405 (0.0891)∗∗∗	0.4226 (0.0855)∗∗∗	0.4798 (0.1018)∗∗∗	0.45 (0.0942)∗∗∗	0.4035 (0.0952)∗∗∗
DIV	0.561 (0.221)∗∗	0.588 (0.2317)∗∗	0.5203 (0.2168)∗∗	0.5595 (0.2244)∗∗	0.7067 (0.3356)∗∗
SIZE	0.0191 (0.0075)∗∗	0.0189 (0.0082)∗∗	0.0211 (0.0084)∗∗	0.0172 (0.007)∗∗	0.0126 (0.0073)∗
GROWTH	0.009 (0.037)	0.0037 (0.0377)	0.0128 (0.0372)	0.0175 (0.0387)	0.0308 (0.0479)
LIQUIDITY	-0.3232 (0.1367)∗∗	-0.3221 (0.1453)∗∗	-0.3433 (0.1435)∗∗	-0.3226 (0.1409)∗∗	-0.1307 (0.207)
INFLATION	0.005 (0.0885)	-0.0124 (0.0941)	-0.0058 (0.0725)	0.0011 (0.0937)	0.0177 (0.1278)
GDPGOWTH	0.0495 (0.1959)	0.0069 (0.2121)	-0.0054 (0.2046)	-0.0004 (0.2205)	0.6525 (0.7599)
Number of obs.	485	485	485	485	485
Wald test (p-value)	0.000	0.000	0.000	0.000	0.000
R-squared	0.9442	0.9454	0.9441	0.9423	0.9349
Root MSE	0.12691	0.12553	0.12692	0.12902	0.13699
Hansen's J test (p-value)	0.3456	0.2319	0.4121	0.3066	0.3315
GMM C statistic (p-value)	0.4153	0.6955	0.3956	0.3062	0.3651

Standard errors in ​brackets. ∗Statistical significance at 10 per cent.∗∗Statistical significance at 5 per cent.∗∗∗Statistical significance at 1 per cent.

**Table 7:** Regression results for Islamicity, bank stability for our sample, and interaction between Islamicity and Islamic banking.

	Column 1: OIS	Column 2: EI	Column 3: LSG	Column 4: HPR	Column 5: IR
Constant	-0.6783 (0.1993)∗∗∗	-0.7468 (0.2019)∗∗∗	-0.7039 (0.1963)∗∗∗	-0.6936 (0.1988)∗∗∗	-0.7474 (0.1985)∗∗∗
Z-score_t-1_	0.9712 (0.0173)∗∗∗	0.9677 (0.0167)∗∗∗	0.9671 (0.017)∗∗∗	0.9687 (0.0174)∗∗∗	0.9667 (0.0272)∗∗∗
IS	-0.0443 (0.0325)	-0.0362 (0.032)	-0.0272 (0.0247)	-0.0316 (0.0319)	-0.0198 (0.0796)
ISLAMIC	-0.0761 (0.0936)	-0.09 (0.1162)	-0.07 (0.1092)	-0.0836 01143)	-0.0289 (0.2576)
IS x ISLAMIC	0.0174 (0.0198)	0.0176 (0.0208)	0.0166 (0.0217)	0.0232 (0.0269)	0.0107 (0.0672)
EFFICIENCY	-0.0462 (0.0257)∗	-0.0358 (0.0283)	-0.0498 (0.0338)	-0.0592 (0.0304)∗	-0.1069 (0.1276)
LERNER	0.4178 (0.0886)∗∗∗	0.4005 (0.0877)∗∗∗	0.4771 (0.0985)∗∗∗	0.4456 (0.0918)∗∗∗	0.3993 (0.1313)∗∗
DIV	0.6558 (0.267)∗∗	0.7002 (0.3043)∗∗	0.5661 (0.231)∗∗	0.6006 (0.2389)∗∗	0.6324 (0.5354)
SIZE	0.0183 (0.0067)∗∗∗	0.0202 (0.0074)∗∗	0.021 (0.008)∗∗	0.0161 (0.0067)∗∗	0.0129 (0.0113)
GROWTH	-0.0005 (0.0362)	-0.0085 (0.037)	0.021 (0.039)	0.0212 (0.0393)	0.0211 (0.0567)
LIQUIDITY	0.0929 (0.0595)	0.0732 (0.0545)	-0.2596 (.1314)∗∗	-0.2562 (0.1222)∗∗	-0.2422 (0.1309)∗
INFLATION	0.0281 (0.0813)	0.0232 (0.0926)	0.0048 (0.0783)	0.0008 (0.0868)	-0.0422 (0.1603)
GDPGOWTH	0.2687 (0.2211)	0.1252 (0.1923)	-0.0471 (0.2231)	0.0108 (0.2157)	0.3504 (1.1549)
Number of obs.	485	485	485	485	485
Wald test (p-value)	0.000	0.000	0.000	0.000	0.000
R-squared	0.9429	0.9439	0.9429	0.9412	0.9434
Root MSE	0.12827	0.12716	0.12829	0.13025	0.12772
Hansen's J test (p-value)	0.2545	0.1459	0.4770	0.3466	0.2129
GMM C statistic (p-value)	0.2889	0.4621	0.3442	0.2655	0.7941

Standard errors in ​brackets. ∗Statistical significance at 10 per cent.∗∗Statistical significance at 5 per cent.∗∗∗Statistical significance at 1 per cent.

We estimate five regressions, and the results are reported in five columns. Column 1 reports the results for the regression where the main independent variable is the Overall Islamicity Index (OIS); Column 2, Column 3, Column 4 and Column 5 accommodate results for the specifications where the constituent indices are the primary independent variables: Economy Islamicity Index (EI), Legal and Governance Index (LSG), Human and Political Rights Index (HPR), and International Relations Index (IR), respectively.

Despite accounting for a range of control variables, endogeneity remains a serious concern (Bilgin et al., 2021). Our estimation may have omitted variables that might affect both Islamicity and bank stability. At the bottom of the table, we report the statistics for each regression and the specification test results for the 2SLS estimations related to endogeneity and over-identification. The results show higher values for the R-squared and that estimations are appropriately specified and free of endogeneity issues or overidentification. GMM C statistic (p-value), endogeneity test, reveals that the employed instruments are valid and jointly relevant to explain the independent variables. The Hansen J-statistic, an overidentification test, reveals that instruments are not correlated with the error term.

Tables 5, 6, and 7 show that our main variables of interest, the Islamicity index and dimensions, are associated with positive coefficients indicating that an increase in Islamicity increases banks' financial resilience. However, the coefficient indicates that the impact of Islamicity is statistically insignificant. This finding suggests that the degree of Islamicity of the environment where banks operate have a neutral effect on the stability of banks regardless of their banking Islamic and conventional model.

Indeed, the central motivation for this paper is to ascertain whether, under current conditions, the level of *Islamicity* of the environment in the dual banking system has consequences on Islamic and conventional banks’ stability. Exploring how Islamic banks behave, the dummy variable "*ISLAMIC*" is consistently and statistically insignificant in all regressions. This suggests that there is no difference between Islamic and conventional banks in terms of financial stability.

Our results are reasonably congruent with some evidence in the literature. For instance, Noland (2005) shows that affiliation with the Islamic faith in Ghana is correlated with economic performance, but the regression estimations fail to generate significantly supporting coefficients. Noland (2005) claims that this result cannot be presented as proof for the no effect of Islamic religious orientation on economic growth. Instead, the result may only be sensitive to the data and periods used. Pryor (2007) shows that the presence of Islamic teachings and practices such as redistribution of income through zakāt, prohibition of *riba* and forbidding of *sinful* activities and excessively risky contracts and ventures has relatively little influence on most economic or social performance indicators. In the analysis of Muslim economies, Islamic teachings and practices do not appear to be a valid explanatory variable. Economic institutions or governmental policies may not have embraced Islamic injunctions. However, Kanagaretnam et al. (2015) find that banks in more religious countries, including Muslim and non-Muslim dominated countries, exhibit less risk-taking in their decision-making. This study uses WVS-sourced person-based measures of religiosity such as Member of Religion, Religion Importance, Religion Services, and Religiosity.

In all regressions, the lagged dependent variable is positive and significant, with a coefficient value of around 0.57, confirming a high degree of persistence in bank stability. The lagged dependent variable and bank-level variables are not entirely exogenous as they are influenced by the banks' managerial discretion (Bilgin et al., 2021). Past information about bank stability in the countries under consideration influences the current level of bank stability.

For the bank-specific control variables, we observe significant coefficients for the variables measuring size, assets growth, liquidity, cost efficiency, competition and diversification, but insignificant coefficient for the variable proxying assets growth. We also observe insignificant coefficients for the variables proxying economic conditions: GDP growth and inflation. These results show that the stability of banks in our sample countries during the period under study is highly influenced by bank-level dichotomies rather than by institutional and environmental conditions.

First, SIZE positively affects banks' stability, meaning larger banks exhibit stronger financial resilience against adverse shocks. Larger banks may be able to enhance their profitability-generating activities and raise equity to reinforce the capitalisation buffer.

Second, the LERNER index of market power as a proxy of competition positively and significantly impacts the stability of banks, implying that the stability of banks in dual-banking markets increases with an increase in their market power. This finding supports the 'competition-fragility' hypothesis, which states that higher competition engenders detrimental effects on bank stability (Boyd and De Nicolo, 2005; Schaeck and Čihák, 2014). It also supports prior studies that the presence of Islamic banks in a banking system intensifies competition, which may erode stability (Kabir and Worthington, 2017; Albaity et al., 2019; Saif-Alyousfi et al., 2020; Risfandy et al., 2010).

Regarding other bank-level controls, we observe a positive coefficient for diversification but negative coefficients for efficiency and liquidity. Banks with better diversification traits are more stable. Banks that generate more income from their noninterest activities are more stable. Our result is consistent with two views. First, the noninterest activities might experience expansion. Second, the traditional interest income activities might suffer shrinking and fluctuations due to the historically lower interest rates and interest margins. This result contrasts with Kohler (2015) and Bilgin et al. (2021), who find that banks with higher income diversification are riskier.

Further, we observe that cost efficiency negatively affects bank stability, meaning less efficient banks are more stable than more efficient banks. This result may capture the fact that for banks to attain higher stability, they may engage in activities that incur higher costs, especially information costs. In addition, banks may be in their expansion phase and have more incentives to incur additional costs to attain other goals, such as expanding market share and integrating the more recent technological advances in the industry known as *Fintechs*. While our result agrees with Kashif et al. (2016), who find a significant negative relationship between efficiency and solvency in Pakistani banking, it contradicts Bilgin et al. (2021), who report a positive efficiency-stability link. Less efficient banks might experience lower profitability and have higher incentives for risky behaviour.

Additionally, we observe that banks with higher growth are more stable. However, the coefficient on the assets growth variable is not statistically significant. Kohler (2015) finds that banks with higher assets growth are riskier because they reduce their lending standards and collateral requirements. Moreover, liquidity is negatively related to stability. Banks with higher liquidity ratios may not be able to allocate liquidity to income-generating activities. Bhowmik and Sarker (2021) argue that the abundance availability of liquid assets as in the GCC banking systems may explain the negative liquidity-risk nexus. This excessive availability resembles less investment by the bank due to the lack of more investment opportunities, resulting in less income. The GCC countries have witnessed a large influx of liquidity due to oil exports and financial inflows, showing that banks had more liquidity than the real economic needs.

About the country controls, higher GDP growth and inflation enhance bank stability. Nevertheless, the coefficients are statistically insignificant. Bilgin et al. (2021) find a positive impact of inflation on bank risk because higher inflation is associated with deteriorating economic conditions. Higher economic growth reflects better economic conditions, which benefit bank stability. Kanagaretnam et al. (2015) find that banks in countries with higher per capita GDP show lower risk levels.

### 5.3 Effect of Islamicity on the stability of Islamic banks

To shed more light on the impact of *Islamicity* in the relationship between Islamic banks and bank stability, we extend our baselines regressions by considering the interaction terms between the proxies of Islamicity and the "*ISLAMIC*" dummy variable. Table 7 shows the regression results using Eq. (7) which focuses on the interaction terms between ISLAMIC and the Islamicity index (IS) and its dimensions.

The coefficients on the interaction terms "*IS x ISLAMIC*" are positive but insignificant in all regressions. This finding implies that the degree of Islamicity, as captured by the overall Islamicity Index and its sub-indices constituents, has a neutral impact on the stability of sharia-compliant banks. The presence of Islamic banks' in dual markets may not specifically be supported by an environment highly consistent with Sharia.

This finding shows that *Sharia*-compliant banks can operate in an environment that is not necessarily Sharia-compliant, and the degree of Islamicity of an environment is neutral in affecting the stability of Islamic banks. Islamic banks may have adapted their *modus operandi* to an environment that is not necessarily compliant with *Sharia* dealings. This result confirms the finding of Chong and Liu (2009) that the practices of the profit-loss sharing model of Islamic banking do not significantly differ from conventional banking. Our results are consistent with Bektas et al. (2022), which suggest that religious orientation has an insignificant influence on bank stability in Islamic-conventional dual banking systems. Bektas et al. (2022) show that the Sharia-based legal system or operating in a Multim-majority country has no significant effect on Islamic (and conventional) banks’ stability.

Our results pinpoint two conclusions. First, Islamic banks' operations might not be entirely compatible with *Sharia*'s framework of *Maqasid Sharia* and *alfalah*. Since Islamic banks operate and compete against conventional banks in an environment dominated by conventional conditions, they are compelled to enter the input and output market relying on conventional parameters to index and set their prices and fees. Second, the variable measuring degree of compatibility of the environment with Sharia principles may not be a perfect measure in capturing the level of Islamicity of the environment (Abdeldayem and Aldulaimi, 2018). This result may be presented as counter-evidence for the claim that Islamic banks may perform better if the environment where they operate is more Sharia-compliant.

## 6 Conclusion

This paper examines the role of a set of bank-level and environmental control variables on the stability of Islamic and conventional banks in countries where the two types work alongside one another. We focus on the role of the *Islamicity* of the environment on bank stability. We test whether Islamic banks are more stable if they operate in an environment characterised by greater compliance with Sharia principles.

*Islamicity* of an environment is congruent with the *alfalah-maqasid Sharia* framework enshrined in *Sharia* and is hypothesised to discipline the risk-taking incentives and influence stability of Islamic banks. The constructs propagated by the *alfalah-maqasid Sharia* framework are closely linked to effectuating the commitment and increasing the propensity of the individuals and organisations to behave well towards others. This will result in ascertaining expectations, reducing costs of obtaining information and reducing information asymmetries. Subsequently, individuals and organisations, including banks, will make more sound decisions.

We use a sample of 51 Islamic and 172 conventional banks from 14 countries during the 2016–19 period. We employ the Islamicity Index to proxy the level of *Islamicity* of an environment and the Z-score to measure banks' stability. Our findings show that Islamic banks do not enjoy superior stability levels than conventional banks. A higher level of *Islamicity* of the environment does not particularly promote Islamic banks' stability in dual banking systems. Hence, the *Islamicity* of the environment is not a significant factor for bank stability, both conventional and Islamic.

Furthermore, our results find a significant impact of bank-level heterogeneities on bank stability: bank size, cost efficiency, competition, income diversification, liquidity, and asset growth.

Our findings support Kuran's view (1993) that newly-established Islamic banks' stability face the intense competition of survivalist character from the ancient conventional banks to the point that they follow their practices and use them as a reference for their pricing and non-pricing activities. Islamic banks may have pursued goals and employed practices and business models separately from the goals and practices embedded in the *Sharia*. Islamic banks seem to be well entrenched in the operational approaches linked to the conventional goal of "*shareholders' wealth maximisation*". In many Muslim countries, sharia-compliant injunctions may not be overwhelmingly incorporated into economic institutions or governmental policies.

The results have several implications for policy and future research. Regulators and supervisors should reconsider the conditions of operations of Islamic banks in a dual banking market. Islamic banks may not be operating in a more Islamised way to differentiate their stability traits from conventional banks. The result supports the suggestion of Chong and Liu (2009) that policy should treat Islamic banks similarly to their conventional counterparts.

Finally, this is a complex area of study requiring a close examination of religiosity measures that can influence banks outcomes across countries and time. Our analysis reveals the need to develop more robust proxies for the environment's degree of religiosity and Islamicity for future research. The reliance on the self-declaration of customers' religious beliefs (Baele et al., 2014) may not be a robust proxy of the Islamicity of an environment. In this regard, the *Maqasid Sharia approach* and *alfalah goal* may offer a solid framework for developing such indices.

## Declarations

### Author contribution statement

Abderazak Bakhouche, Teheni El Ghak: Conceived and designed the experiments; Performed the experiments; Analyzed and interpreted the data; Contributed reagents, materials, analysis tools or data; Wrote the paper.

Mohammad Alshiab: Conceived and designed the experiments.

### Funding statement

This research did not receive any specific grant from funding agencies in the public, commercial, or not-for-profit sectors.

### Data availability statement

Data will be made available on request.

### Declaration of interests statement

The authors declare no conflict of interest.

### Additional information

No additional information is available for this paper.

## Footnotes

1 Abu Ḥamid Muḥammad ibn Muḥammad aṭtūsiyy Al-Ghazzali (1058–1111), Al-Ghazzli extended a doctrine of Islamic legal jurisprudence mounted earlier by his teacher Abu Al-Maali Al-Jowayni (1028–1085).

2 Muhammad al-Ṭāhir ibn ʿĀshūr (1879–1973): The Foundations of the Social system in Islam.

3 Berger et al. (2009), Shaddady and Moore (2019) and Al-Shboul et al. (2020) find that institutional environments significantly affect bank risk-taking.

4 For further details on the indicators used to construct elements, please see “Islamicity Indices and their Elements” available at: http://islamicity-index.org/wp/links-downloads/index-elements/.
